# Preoperative Pressure Ulcers, Mortality, and Complications in Older Hip Fracture Surgery Patients

**DOI:** 10.5435/JAAOSGlobal-D-22-00117

**Published:** 2022-11-02

**Authors:** Steven B. Porter, Raymond Pla, Jonathan H. Chow, Ryan Keneally, Rundell Douglas, Tricia Desvarieux, Matthew M. Crowe, Michael A. Mazzeffi

**Affiliations:** From the Department of Anesthesiology and Perioperative Medicine, Mayo Clinic, Jacksonville, FL (Dr. Porter); the Department of Anesthesiology and Critical Care Medicine, George Washington University School of Medicine and Health Sciences, Washington, DC (Dr. Pla, Dr. Chow, Dr. Keneally, Dr. Desvarieux, and Dr. Mazzeffi); the Milken Institute School of Public Health, George Washington University, Washington, DC (Douglas); and the Department of Orthopedic Surgery, Mayo Clinic, Jacksonville, FL (Dr. Crowe).

## Abstract

The effect of a preoperative pressure ulcer (PPU) in hip fracture patients on postoperative outcomes has not been well studied. We hypothesized that the presence of a PPU would be associated with increased mortality and serious complications in hip fracture surgery patients.

**Methods:**

We conducted a cohort study of 19,520 hip fracture patients from 2016 to 2019 with data from the National Surgical Quality Improvement Program. The study exposure was the presence of a PPU. This study's primary outcome was 30-day mortality. Secondary outcomes included deep vein thrombosis (DVT), pulmonary embolism, surgical site infection, pneumonia, and unplanned hospital readmission. Propensity score analysis and inverse probability of treatment weighting were used to control for confounding and reduce bias.

**Results:**

The presence of a PPU was independently associated with a 21% increase in odds of 30-day mortality (odds ratio (OR) = 1.2, *P* = 0.004). The presence of a PPU was also independently associated with increased odds of DVT (OR = 1.59, *P* < 0.001), pneumonia (OR = 1.39, *P* < 0.001), and unplanned hospital readmission (OR = 1.43, *P* < 0.001) and a significant increase in the mean length of hospital stay of 0.4 days (*P* = 0.007).

**Discussion:**

We found that PPUs were independently associated with increased 30-day mortality, DVT, pneumonia, hospital length of stay, and unplanned hospital readmission.

Hip fractures in older Americans continue to represent a major public health problem representing 10% to 15% of approximately two million osteoporotic fractures in the United States while accounting for nearly 75% of all hip fracture costs.^1,2^ Many patients have notable comorbidities that would otherwise preclude them from elective orthopaedic surgery. Surgery is recommended, even in palliative situations, to allow for mobilization and pain control. Despite improvements in perioperative medicine, many candidates for hip fracture surgery remain at high risk of morbidity and mortality, with an estimated one-year mortality of 20% to 30%.^3^

Strategies to manage medical comorbidities and surgical variables have been created to help coordinate care for hip fracture surgery patients.^4,5^ The American Academy of Orthopaedic Surgeons has also issued consensus guidelines for the management of hip fractures in the elderly.^6^ These include completing surgery within 48 hours when possible, not delaying surgery for patients on aspirin and/or clopidogrel, recommendations for postoperative deep vein thrombosis (DVT) prophylaxis, and outpatient osteoporosis workup. One factor that is not modifiable at the time of presentation is the presence of a preoperative pressure ulcer (PPU). PPUs are associated with increased complications and mortality in general surgical patients.^7^ However, little is known about their effect on mortality and serious complications in older hip fracture surgery patients. Our goal was to study the association between PPUs and in-hospital mortality. We hypothesized that the presence of a PPU at admission would be independently associated with increased odds of mortality.

## Methods

### Patients

The Institutional Review Board at the Mayo Clinic approved this study and granted a waiver of written informed consent. Adults who had hip fracture surgery were identified using the 2016 to 2019 National Surgical Quality Improvement Program (NSQIP) Participant Use Files (PUFs). Specifically, we included patients who had Current Procedural Terminology (CPT) codes 27236 (open treatment of femoral fracture, proximal end, neck, internal fixation, or prosthetic replacement) and 27245 (treatment of intertrochanteric, peritrochanteric, or subtrochanteric femoral fracture with intramedullary implant).

Study exclusions were preoperative sepsis, age younger than 75 years, body mass index (BMI) less than 16.5 kg/m^2^ or greater than 50 kg/m^2^, duration of surgery greater than the 99th percentile or less than the first percentile, preoperative mechanical ventilation, and disseminated cancer. We used these exclusions to increase the likelihood of good covariate balance between patients who did and did not have a PPU. Patients were also excluded if they were missing data for a variable in the propensity score model.

### Study Data

Patients had demographics, medical comorbidities, and surgical details collected. Definitions for all study variables were according to NSQIP (www.facs.org). The following variables were collected: age, sex, race, BMI, location admitted from, hematocrit, platelet count, creatinine, international normalized ratio, albumin, baseline functional status, diabetes mellitus, hypertension requiring medication, congestive heart failure, tobacco use, chronic obstructive pulmonary disease, dialysis, dementia, wound classification, steroid use, malnourishment, red blood cell transfusion in the 72 hours before surgery, emergency surgery, American Society of Anesthesiologists (ASA) physical status classification, predicted mortality, anesthesia type, CPT code, and total surgical minutes.

### Exposure

The study exposure was the presence of a PPU. NSQIP does not collect data on the stage of pressure ulcer that is present; thus, the pressure ulcer stage was not analyzed.

### Outcomes

This study's primary outcome was 30-day mortality. Secondary outcomes included DVT, pulmonary embolism, surgical site infection (superficial and deep), pneumonia, and unplanned hospital readmission.

### Statistical Analysis

Statistical analysis was conducted using SAS 9.3 (SAS). The Strengthening the Reporting of Observational Studies in Epidemiology checklist was referenced in designing this study and reporting its results. Patients were stratified by whether they had a PPU. Continuous variables were summarized as mean + standard deviation, and categorical variables were summarized as the number (%) of patients. Covariate balance was assessed between the two groups by calculating standardized mean differences (SMDs). Notable covariate imbalance was indicated by SMDs that were greater than 0.2.

To reduce bias, propensity score analysis and inverse probability of treatment weighting (IPTW) were conducted. For the propensity score model, the presence of a PPU at the time of surgery was modeled as the dependent variable. Independent variables in the model included all demographics, comorbidities, and surgical details that might affect the risk of having a pressure ulcer or 30-day mortality. Specific variables that were included in the model were age, sex, race, BMI, location admitted from, hematocrit, platelet count, creatinine, baseline functional status, diabetes mellitus, hypertension requiring medication, congestive heart failure, tobacco use, chronic obstructive pulmonary disease, dialysis, dementia, wound classification, steroid use, malnourishment, red blood cell transfusion in the 72 hours before surgery, emergency surgery, ASA physical status classification, predicted mortality, anesthesia type, CPT code, and total surgical minutes.

IPTWs were calculated for each patient using propensity scores, and IPTW adjustment was used to balance covariates between patients who had a PPU and those who did not. IPTW allows for estimation of the causal effect when measured covariates are balanced between groups.^8^ After IPTW adjustment, covariate balance was again assessed using SMDs. To calculate the average treatment effect of having a PPU on 30-day mortality and secondary outcomes, IPTW-adjusted logistic regression was used. Odds ratios (ORs) with 99% confidence intervals (CIs) were calculated to account for multiple statistical tests. A priori sample size calculation was not conducted for this study. All patients available in the database with eligible CPT codes were included.

## Results

**Figure 1:**
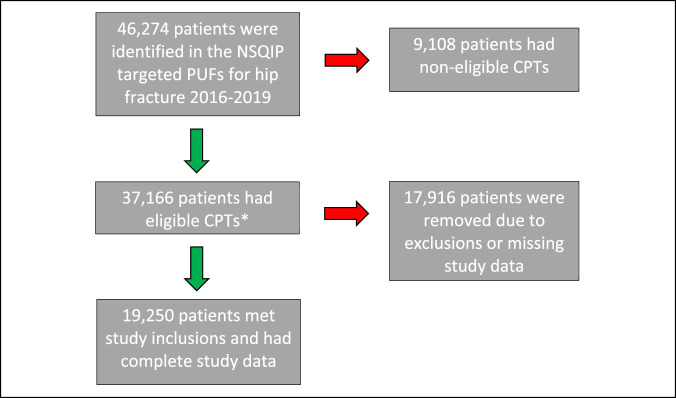
Flow diagram.

Figure 1 shows the study enrollment diagram. There were 37,166 patients in the targeted hip fracture PUFs who had eligible CPTs. Of these, 19,250 met inclusions and had complete study data. Supplemental Table 1 (http://links.lww.com/JG9/A239) summarizes patient characteristics in the cohort before propensity score analysis and IPTW. Notable covariate imbalance was observed between patients who had a PPU and those who did not. Specific covariates that were not well balanced included admission from a nursing home, international normalized ratio, dementia, hematocrit, albumin, independent baseline functional status, congestive heart failure, ASA physical status, and mortality probability (all SMDs ≥0.2).

Supplemental Table 2 (http://links.lww.com/JG9/A240) summarizes covariate balance after propensity score analysis and IPTW. All covariates were well balanced with no covariate having SMD ≥0.2. The mean age of patients in the IPTW cohort was 85 years, and most of the patients were female (71.8%). The prevalence of dementia was high in the cohort at 29.7%. Mean surgical time was 63 minutes

Table 1 summarizes study outcomes with IPTW-adjusted ORs and *P* values. The presence of a PPU was independently associated with a 21% increase in odds of 30-day mortality (OR = 1.21, 99% CI = 1.02 to 1.43, *P* = 0.004). The presence of a PPU was also independently associated with increased odds of DVT (OR = 1.59, 99% CI = 1.15 to 2.20, *P* < 0.001), pneumonia (OR = 1.39, 99% CI = 1.15 to 1.69, *P* < 0.001), and unplanned hospital readmission (OR = 1.43, 99% CI = 1.26 to 1.62, *P* < 0.001). Furthermore, the presence of a PPU was associated with a significant increase in the mean length of stay (0.4 days; 99% CI = 0.02 to 0.8, *P* = 0.007).

**Table 1:** Outcomes After IPTW

Variable	No Pressure Ulcer N = 18,996	Pressure Ulcer N = 524	IPTW OR with 99% CI or mean difference with 99% CI^a^	*P* value^b^
30-day mortality	893 (4.7)	29 (5.6)	1.21 (1.02-1.43)	0.004
DVT	209 (1.1)	9 (1.7)	1.59 (1.15-2.20)	<0.001
PE	133 (0.7)	3 (0.6)	0.76 (0.48-1.21)	0.13
SSI—superficial	95 (0.5)	2 (0.3)	0.62 (0.35-1.10)	0.03
SSI—deep	19 (0.1)	1 (0.2)	1.82 (0.75-4.43)	0.08
Pneumonia	627 (3.3)	24 (4.5)	1.39 (1.15-1.69)	<0.001
Length of hospital stay	5.7 + 6.7	6.1 + 51.3	0.4 (0.02-0.8)^c^	0.007
Unplanned readmission	1539 (8.1)	58 (11.1)	1.43 (1.26-1.62)	<0.001

DVT = deep vein thrombosis, IPTW = inverse probability of treatment weight, OR = odds ratio, PE = pulmonary embolism, SSI = surgical site infection

a ORs are the odds for the outcome if a pressure ulcer is present on admission.

b *P* value for IPTW-adjusted OR.

c This outcome is a mean difference; all others are IPTW ORs.

Values are mean + SD or n (%).

## Discussion

In an observational cohort study of 19,520 hip fracture surgery patients, we found that the presence of a PPU was independently associated with increased 30-day mortality, DVT, pneumonia, hospital length of stay, and unplanned hospital readmission after IPTW. The presence of a PPU was not associated with an increase in pulmonary embolism, nor superficial or deep surgical site infection. Our findings suggest that PPUs should be included in current risk models for morbidity and mortality after hip fracture surgery. Furthermore, PPUs may indicate higher risk of specific postoperative complications, including DVT and pneumonia, and enhanced preventive strategies and surveillance strategies may be indicated in patients with PPU .

To the best of our knowledge, our study is the largest to explore the effect of a PPU on perioperative outcomes in older hip fracture surgery patients. Other researchers have previously investigated the association between PPUs and outcomes in general surgical patients. For example, Chou et al^7^ compared 17,391 adult surgical patients with a PPU with an equal number of surgical patients without a PPU. The study was exclusive to Taiwan, using data from 2008 to 2010, and found that patients with a PPU had markedly higher 30-day postoperative mortality (relative risk 1.83) and pneumonia. Our analysis demonstrated increased 30-day mortality and pneumonia in hip fracture surgery patients with a PPU, but only US patients who had surgery between 2016 and 2019 were included. The study by Chou et al included patients who had multiple surgical procedures under general or neuraxial anesthesia and a hospitalization of ≥1 day. Among 17,391 surgical patients, 7,856 in each group had “musculoskeletal” surgery. The authors reported that 2,104 patients had a comorbid hip fracture, but it is not clear how many patients had hip fracture surgery. Our age criteria (75 years or older) differed drastically from that of Chou et al where any patient 20 years or older was included.

PPUs are a marker of poor nutritional and functional status^9^ and may capture information that is distinct from other described risk factors. For example, Galivanche et al^10^ identified PPU as a risk factor for developing a postoperative pressure ulcer. In their study of 8,871 geriatric hip fracture patients, the development of a postoperative pressure ulcer was associated as a risk factor for the development of postoperative pneumonia, as we also found. PPUs may also be a marker of poor wound healing, which is important in surgical patients. The relationship between PPU and DVT in our study may reflect the severe immobility shared by many patients with a PPU,^11^ which may disproportionately increase their DVT risk. Similarly, pneumonias may be more common in patients with PPU because of reduced muscle mass, poor cough strength, comorbid dementia or cognitive dysfunction, and an inability to be upright in bed.^11^ To account for these chronic comorbidities, we used propensity score modeling and IPTW to balance dementia, BMI, ASA classification, malnourishment, albumin, independent functional status at baseline, and admitted from a nursing home, among other observed risk factors.

Our study has several limitations. First, owing to this study's retrospective nature, diagnoses and outcomes were based on abstractions per NSQIP's standard definitions. Some data may have been abstracted or entered incorrectly, and this could lead to bias in our analysis. Second, the NSQIP hip fracture-targeted PUF does not contain data on the PPU stage, so we were unable to account for whether PPU severity affected outcomes. Third, NSQIP does not contain hospital-level data, and hence, we were unable to control for hospital in our analysis. There may have been residual confounding in our analysis if low-quality hospitals treated more patients with PPUs because low-quality hospitals tend to have more potentially preventable conditions (e.g., DVT, pneumonia).

## Conclusion

In an observational cohort study of 19,520 hip fracture patients, we found that PPUs were associated with increased 30-day mortality, DVT, pneumonia, hospital length of stay, and unplanned hospital readmission. These findings are important so that appropriate risk adjustment can be done for hip fracture surgery patients moving forward. Furthermore, the presence of a PPU may help identify patients at risk of potentially preventable complications such as DVT and pneumonia. Heightened surveillance or more aggressive preventive strategies may be warranted in these patients.
